# Short Antimicrobial Peptides Based on Arginine and Tryptophan: Agents with Potential in Combating Resistant Pathogens

**DOI:** 10.1021/acsomega.5c12724

**Published:** 2026-03-13

**Authors:** Eric Fernández de la Cruz, Jessica T. Mhlongo, Ashish Kumar, Fernando Albericio, Miguel Viñas, Paula Espinal, Ester Fusté, Beatriz G. de la Torre

**Affiliations:** † Laboratory of Molecular Microbiology & Antimicrobials, Department of Pathology and Experimental Therapeutics, Faculty of Medicine & Health Sciences, IDIBELL-University of Barcelona, Campus Bellvitge, L’Hospitalet de Llobregat, Barcelona 08907, Spain; ‡ Medical School, College of Health Sciences, 56394 University of KwaZulu-Natal, Durban, KwaZulu-Natal 4041, South Africa; § Peptide Science Laboratory, School of Chemistry and Physics, University of KwaZulu-Natal, Durban, KwaZulu-Natal 4001, South Africa; ∥ Department of Inorganic and Organic Chemistry, University of Barcelona, Barcelona 08028, Spain; ⊥ Department of Public Health, Mental Health and Maternal and Child Health Nursing, University of Barcelona, Campus Bellvitge, L’Hospitalet de Llobregat, Barcelona 08907, Spain

## Abstract

The limitations of conventional antibiotics due to the rise of antimicrobial resistance demand new therapeutic strategies. Antimicrobial peptides represent a promising alternative because of their broad-spectrum activity and low propensity for inducing resistance. In this study, we designed and evaluated a set of seven-residue Arg/Trp-based peptides. All peptides were successfully synthesized and characterized, and their activities were assessed against *Escherichia coli*, *Pseudomonas aeruginosa*, and *Staphylococcus aureus*, including clinical isolates. Among the sequences tested, (WRW)_2_F exhibited the strongest antimicrobial activity, displaying bactericidal effects. Growth inhibition and time-kill assays showed dose-dependent effects, and SYTOX Green uptake, AFM, and TEM analyses confirmed rapid membrane permeabilisation and structural disruption as the primary mode of action, while it has been demonstrated not to act as an efflux pump inhibitors. Importantly, the three more active peptides synergized with linezolid against *E. coli,* and (WRW)_2_F was able to resensitize MRSA to oxacillin, demonstrating their potential for combined therapies. The toxicity studies in eukaryotic cells and the *C. elegans* survival model have shown a favorable safety profile. Overall, this work highlights short Arg/Trp-rich peptides, especially (WRW)_2_F, as affordable, easy-to-synthesize, and biocompatible compounds with significant potential to enhance antibiotic efficacy and counteract resistant pathogens.

## Graphical abstract

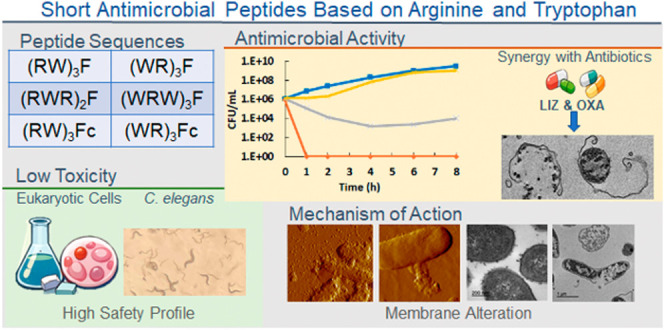

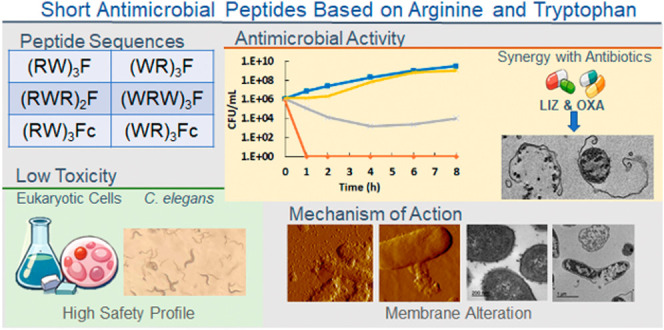

## Introduction

The rapid emergence and spread of antimicrobial-resistant bacteria significantly outpaces the development of new antibiotics and the progress in the use of other antimicrobial weapons such as phage therapy, CRISPR-Cas9, combination of antimicrobials, or exploration of natural compounds.^1,2^ This imbalance seriously threatens global health as infections that were once-treatable become difficult, even impossible, to cure. Therefore, there is an urgent need for effective countermeasures to overcome the risk of entering a postantibiotic era where routine surgeries, cancer treatments, and even minor injuries would carry increased risks due to untreatable infections and could become life-threatening. This global trend is evidenced by the rising mortality rates associated with antimicrobial resistance (AMR), what has become a critical public health concern.^3^ Addressing this escalating crisis requires a multifaceted approach, including the accelerated development of novel therapies, the promotion of responsible use of existing antimicrobials, and the implementation of comprehensive global strategies focused on surveillance, prevention and control, and innovation.^4^ In the last decades, antimicrobial peptides (AMPs) have emerged as potential alternative antimicrobials based mainly on their broad-spectrum activity and low resistance induction.^5^ Some of them have also shown synergistic effects when tested with conventional antibiotics as well as certain capability to modulate the immune response.^6^ In general, AMPs consist of 10–50 amino acids with a high content of basic residues. Thus, they are cationic but also have around 50% hydrophobic amino acids, which confer amphipathic properties that are key in the antibacterial activity.^7^ Arginine and lysine, two side-chain positively charged amino acids, are particularly abundant, representing approximately 20–35% of the residues in many AMPs. These positive charges enhance electrostatic interactions with negatively charged bacterial membranes, while hydrophobic amino acids facilitate insertion into lipid bilayers and contribute to structural instability.^8^ The most prevalent hydrophobic residues in AMPs include leucine, isoleucine, valine, tryptophan, and phenylalanine, accounting for 30–50% of the amino acid composition. In this regard, we have focused our interest in short peptides based on Arg/Trp repeats as this type of sequence has been previously reported to exhibit a favorable balance between antibacterial activity and toxicity, positioning them as promising candidates for further development.^9–14^ The study focuses on seven amino acid peptide length that contains a Phe residue at the C-terminus, as its hydrophobicity would facilitate the insertion into bacterial membranes, and includes the following designs: (i) three pairs of RW or WR to study the influence of amino acid order; (ii) two triads, RWR and WRW, to evaluate the effect of different balances between positively charged and hydrophobic residues; and (iii) a disulfide cyclic peptide version of (i) by adding Cys at both ends (Table 1). Usually, constricted peptides, such as cyclic peptides, show better stability and activity than their linear counterparts. It is a common practice to use disulfide bond for cyclization purposes.

**Table 1:** Peptides Included in the Study

**peptide**	**sequence**	**molecular mass**	**charge**	**hydrophilicity**
(RW)_3_F	RWRWRWF	1191.42	+4	–0.53
(WR)_3_F	WRWRWRF	1191.42	+4	–0.53
(RWR)_2_F	RWRRWRF	1161.39	+5	0.39
(WRW)_2_F	WRWWRWF	1221.44	+3	–1.44
(RW)_3_Fc	CRWRWRWFC	1437.72	+3	–0.63
(WR)_3_Fc	CWRWRWRFC	1437.72	+3	–0.63

## Results and Discussion

### Synthesis of Peptides

Peptides were obtained as amides at the C-terminus and with free N-termini. They were synthesized using solid-phase methodology by standard Fmoc/tBu strategy. All peptides were purified to homogeneity >95% and characterized by mass spectrometry before biological testing (Figures S1–S6). A previous study on (RW)_*n*_ peptide series, being *n* = 1 to 5, demonstrated that the minimum number of RW to have antibacterial activity was three. Although the potency of the peptide increased as n increased, the hemolytic effect was higher.^11^ Thus, (RW)_3_ was used as a starting sequence, to which a Phe residue was added to the C-terminus, as its hydrophobicity would facilitate penetration into bacterial membranes.^15^ The following parameters have been studied: (i) the importance on the sequence order by testing (RW)_3_F vs (WR)_3_F; (ii) the increase and decrease of hydrophobicity, (RWR)_2_F and (WRW)_2_F; and (ii) the effect of cyclization through two extra Cys residues added at both ends.^16^

### Antimicrobial Activities of Peptides

The MIC (minimum inhibitory concentration) of the compounds was determined against *Escherichia coli*, *Pseudomonas aeruginosa* and *Staphylococcus aureus* in both collection and clinical isolates (Table 2). In the case of (RW)_3_F and (WR)_3_F the MIC values obtained were similar, suggesting a nonsignificant influence of the sequence order in the case of these two peptides that share the number of positive charges and hydrophilicity value (Table 1). Interestingly, the most hydrophobic peptide (WRW)_2_F, with a lower net positive charge (+3) than the other linear peptides, showed the highest antimicrobial activity against both *E. coli* and *S. aureus*.

**Table 2:** MIC of Peptides against Control and Clinical Strains

**MIC (μg/mL)**						
**isolate**	**(RW)**_**3**_ **F**	**(WR)**_**3**_ **F**	**(RWR)**_**2**_ **F**	**(WRW)**_**2**_ **F**	**(RW)**_**3**_ **Fc**	**(WR)**_**3**_ **Fc**
*E. coli* ATCC 25922	32	32	>64	16	>64	>64
*E. coli* 208691	>64	>64	>64	16	>64	>64
*P. aeruginosa* ATCC 27853	64	32	>64	64	>64	>64
*P. aeruginosa* 666	>64	>64	>64	64	>64	>64
*S. aureus* ATCC 29213	>64	64	>64	8	>64	>64
*S. aureus* 11^a^	>64	>64	>64	16	>64	>64
*S. aureus* 19	>64	>64	>64	16	>64	>64
						

a MRSA: methicillin resistant *S. aureus*.

As expected, the lower antimicrobial power was displayed in *P. aeruginosa*, a species characterized by the low permeability of its outer membrane and by the intrinsic resistance determinants. This reduced susceptibility may be attributed to the limited permeabilization capacity of peptides with lower net charge or to the barrier function of the lipopolysaccharide (LPS) layer, which can hinder peptide access to the inner membrane.^11,17^ The poor activity of other RW peptides against *P. aeruginosa* has been reported previously.^18^ On the other hand, (RWR)_2_F, the peptide with the higher positive net charge and less hydrophobicity, showed a lower potency against all the strains tested, with all MICs > 64 μg/mL.

These findings are consistent with those reported by Jindal et al. and Sunda-Meya and Phambu et al., highlighting the critical role of balancing charge and hydrophobicity to optimize bacterial membrane interaction and disruption.^18,19^ Nevertheless, the precise relationship between net charge and antimicrobial activity remains unclear.^18^ A similar trend was observed upon cyclization via disulfide bond formation, achieved by introducing two cysteine residues at both termini of the peptide. Based on these findings, the three compounds (RW)­3F, (WR)­3F, and (WRW)­2F were selected for further study.

To gain deeper insight into the antibacterial activity of the peptides, growth kinetic experiments were conducted in the presence of (RW)_3_F, (WR)_3_F and (WRW)_2_F (Figure 1). Bacteria were incubated in the presence of inhibitory and subinhibitory concentrations (MIC, 1/2 MIC, and 1/4 MIC) of the peptides. In *E. coli* ATCC 25922 and *P. aeruginosa* ATCC 27853, all three peptides completely inhibited the growth over 24 h period when treated at MIC as expected. Moreover, at 1/2 MIC, a substantial delay in growth was observed compared to the control, while at 1/4 MIC, slight inhibition of growth was noted, indicating that this concentration is close to or below the minimum required to significantly impair bacterial proliferation.

**Figure 1:**
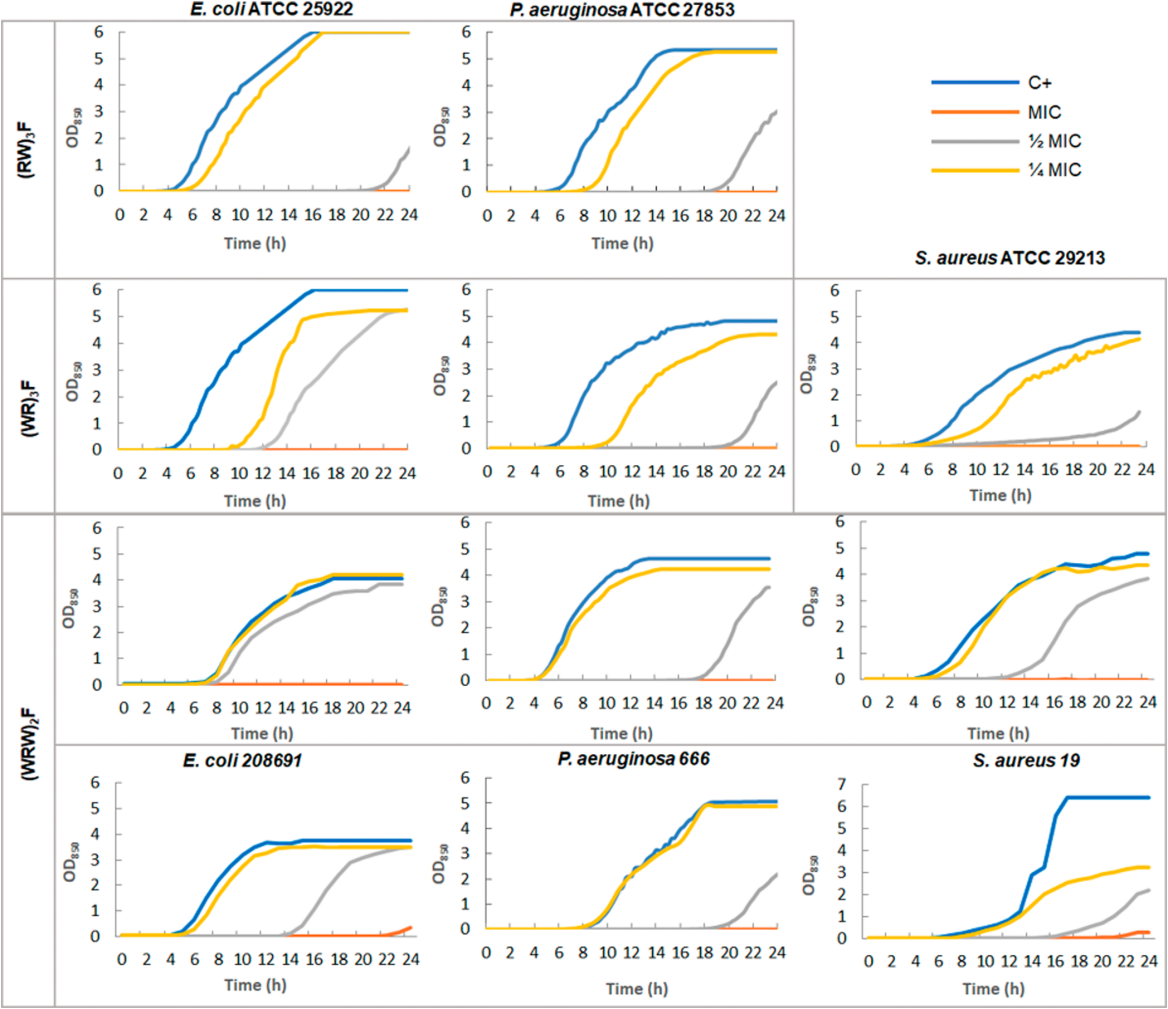
Growth curves of different bacterial strains in the presence of (RW)­3F, (WR)­3F, and (WRW)­2F at MIC (orange line), 1/2 MIC (gray line), and 1/4 MIC (yellow line).

In *S. aureus* ATCC 29213, (WR)­3F, and (WRW)­2F gave comparable results, achieving complete growth inhibition within 24 h. Among the peptides tested, (WRW)_2_F exhibited the most potent antimicrobial activity against *S. aureus*, showing pronounced bacterial growth inhibition at substantially lower concentrations compared to the other compounds.

These findings underscore the relevant antimicrobial activity of the peptides at subinhibitory concentrations, mainly at 1/2 MIC, as evidenced by the delayed growth observed in the bacteria tested. A recent study published by Berryhill et al. also emphasizes the importance of the effects of sub-MIC concentrations on bacterial growth dynamics, showing that they can delay the onset of bacterial proliferation and reduce growth rate.^20^ This suggests that exposure to antimicrobials at sub-MIC levels may compel bacteria to allocate more resources to essential biological processes, such as replication. Modifying administration guidelines could be useful in situations where the MIC concentration in tissues cannot be achieved.

However, low concentrations should be combined with other antimicrobials to avoid the harmful effect they could have on the emergence of resistant forms.^21^

Subsequently, time-kill kinetic assays were performed to determine whether the active compounds showed time-dependent or concentration-dependent effects on bacteria, and whether these effects resulted in a bacteriostatic or bactericidal mode of action. The time-kill kinetics of (RW)_3_F in *E. coli* demonstrated that the peptide does not induce significant bacterial death at MIC or 1/2 MIC. At 1/4 MIC, CFU/mL remained comparable to the control throughout the assay. In the case of *P. aeruginosa*, the time-kill curve indicates that a certain level of bacterial death occurs at the MIC, whereas, as in the case of *E. coli*, subinhibitory concentrations fail to efficiently reduce viable cell counts over time (Figure 2, upper panel). This is consistent with the bacteriostatic behavior of this peptide.

**Figure 2:**
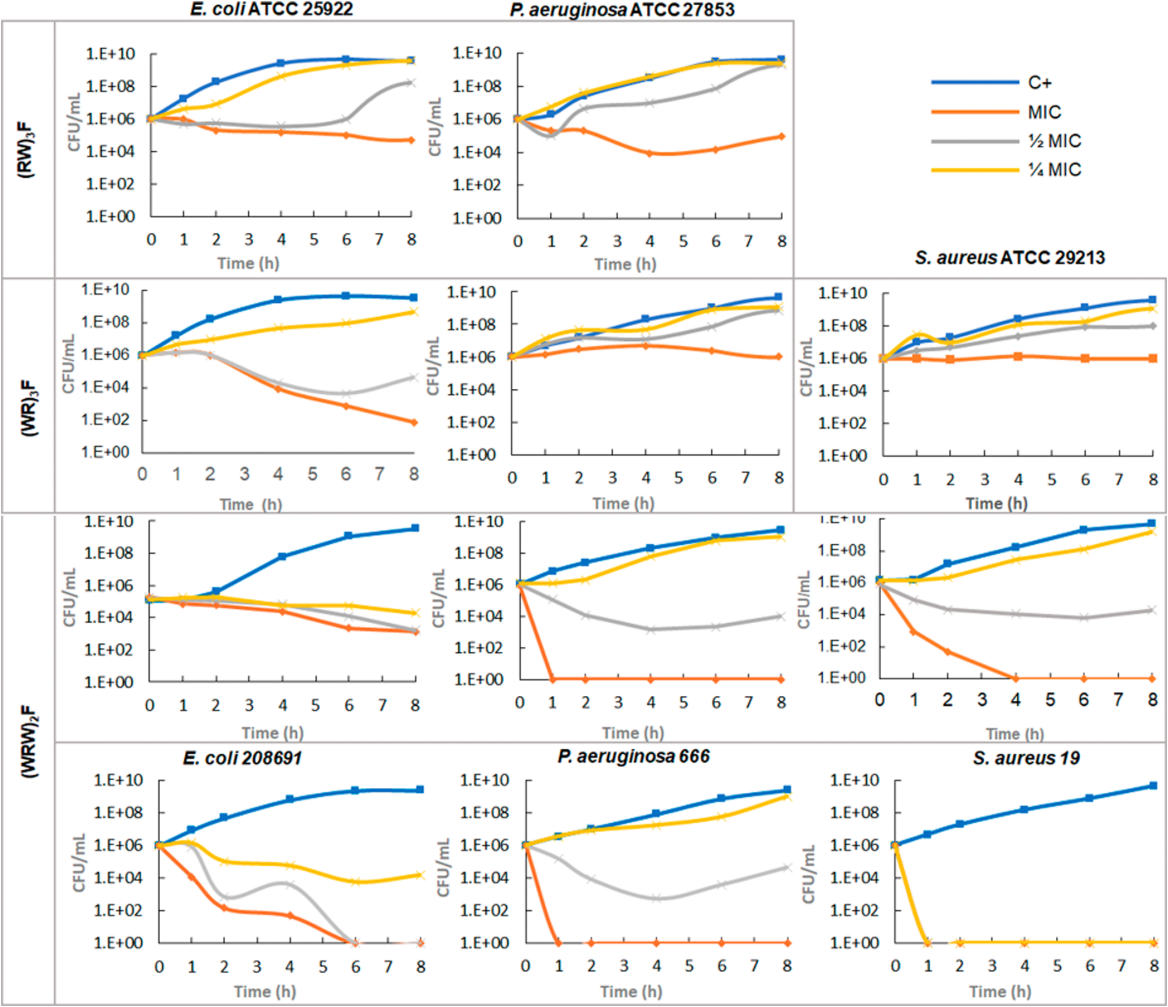
Time-kill curves of different bacterial strains in the presence of (RW)­3F, (WR)­3F, and (WRW)­2F at MIC (orange line), 1/2 MIC (gray line), and 1/4 MIC (yellow line).

In the case of (WR)_3_F, a bacteriostatic effect was also observed in *P. aeruginosa* and *S. aureus* when treated at the MIC, with a lesser effect on bacterial death kinetics at subinhibitory concentrations. However, in *E. coli*, the exposure to (WR)_3_F at MIC resulted in a gradual decline in viable cell counts beginning after 2 h, ultimately reaching a 4-log reduction after 8 h of treatment. Additionally, a similar trend was observed at 1/2 MIC, although regrowth occurred sooner (6 h) (Figure 2, central panel).

In contrast to the bacteriostatic effect shown by the former peptides, (WRW)_2_F showed a bactericidal effect on both ATCC and clinical strains of *P. aeruginosa* and *S. aureus* at MIC. In *E. coli*, the antimicrobial activity of this peptide varied among the strains studied, further research is required to determine the underlying reason for this difference which, interestingly, yields a result in which it performs better against the pathogen than against the collection strain. (Figure 2, lower panel).

The results of the antimicrobial activity indicate that peptide (WRW)_2_F exhibits a markedly higher antimicrobial effect compared to (RW)_3_F and (WR)_3_F. This was particularly evident in *E. coli* and *S. aureus*. These findings highlight the potent nature of (WRW)_2_F as an antimicrobial agent.

### Interaction with Conventional Antibiotics

The combination of AMPs and conventional antibiotics to defeat bacterial infections has gained attention as their clinical potential is based in two principles: repurposing of antibiotics by broadening their spectrum of activity and resensitizing antibiotic-resistant strains.^5,18,22^ Table 3 presents the results of the synergistic effects of the peptides in combination with linezolid and oxacillin. The combination of peptides (RW)_3_F, (WR)_3_F, and (WRW)_2_F with linezolid was evaluated against the Gram-negatives *E. coli* ATCC 25922 and *P. aeruginosa* 27853. Additionally, considering that the peptide (WRW)_2_F exhibited the strongest antimicrobial activity against *S. aureus*, including MRSA, we investigated its potential to resensitize MRSA to β-lactam antibiotics by combining it with oxacillin.

**Table 3:** Interaction between Antimicrobial Peptides and Conventional Antibiotics^a^

	**FICi**		
bacterial strain	**[(RW)**_**3**_ **F, LIZ]**	**[(WR)**_**3**_ **F, LIZ]**	**[(WRW)**_**2**_ **F, LIZ]**
*E. coli* ATCC 25922	0.25 Synergistic	0.25 Synergistic	0.375 Synergistic
*P. aeruginosa* ATCC 27853	1.5 No interaction	1.5 No interaction	2 No interaction

	**[(RW)**_**3**_ **F, OXA]**	**[(WR)**_**3**_ **F, OXA]**	**[(WRW)**_**2**_ **F, OXA]**
*S. aureus* 11 (MRSA)	NA	NA	0.5 Synergistic

a FICi: Fractional inhibitory concentration index; Interpretation: synergistic FICi ≤ 0.5; No interaction FICi > 0.5–4. NA = Non applicable; MRSA: methicillin resistant *S. aureus*; LIZ = linezolid; IMI = imipenem; OXA = oxacillin.

A synergistic effect of the three peptides in combination with linezolid was detected for *E. coli*, with FICi values ranging from 0.25 to 0.375; however, no effect was observed in *P. aeruginosa* (Table 3). Interestingly, a synergistic effect was observed against *S. aureus* 11 (MRSA), suggesting that (WRW)_2_F may potentially resensitize the strain to oxacillin when used in combination.

Growth kinetics experiments were conducted exclusively on bacterial strains and peptide combinations for which synergistic interactions were previously identified, to provide complementary insights into the dynamics of bacterial inhibition under these conditions (Figure 3A). A noticeable reduction in *E. coli* growth was observed when the peptides were used at a concentration of 1/8 MIC combined with linezolid at concentrations ranging from 1/8 MIC to 1/4 MIC (Figure 3A), while linezolid alone was inactive as was the peptide at the concentrations tested. The results were similar for all three peptides, although (WRW)­2F maintained inhibition only up to 12 h, while the other two inhibited growth completely for 24 h. This result opens up new horizons in the design of treatments for Gram-negative bacteria using relatively low concentrations of linezolid, despite their intrinsic resistance.

**Figure 3:**
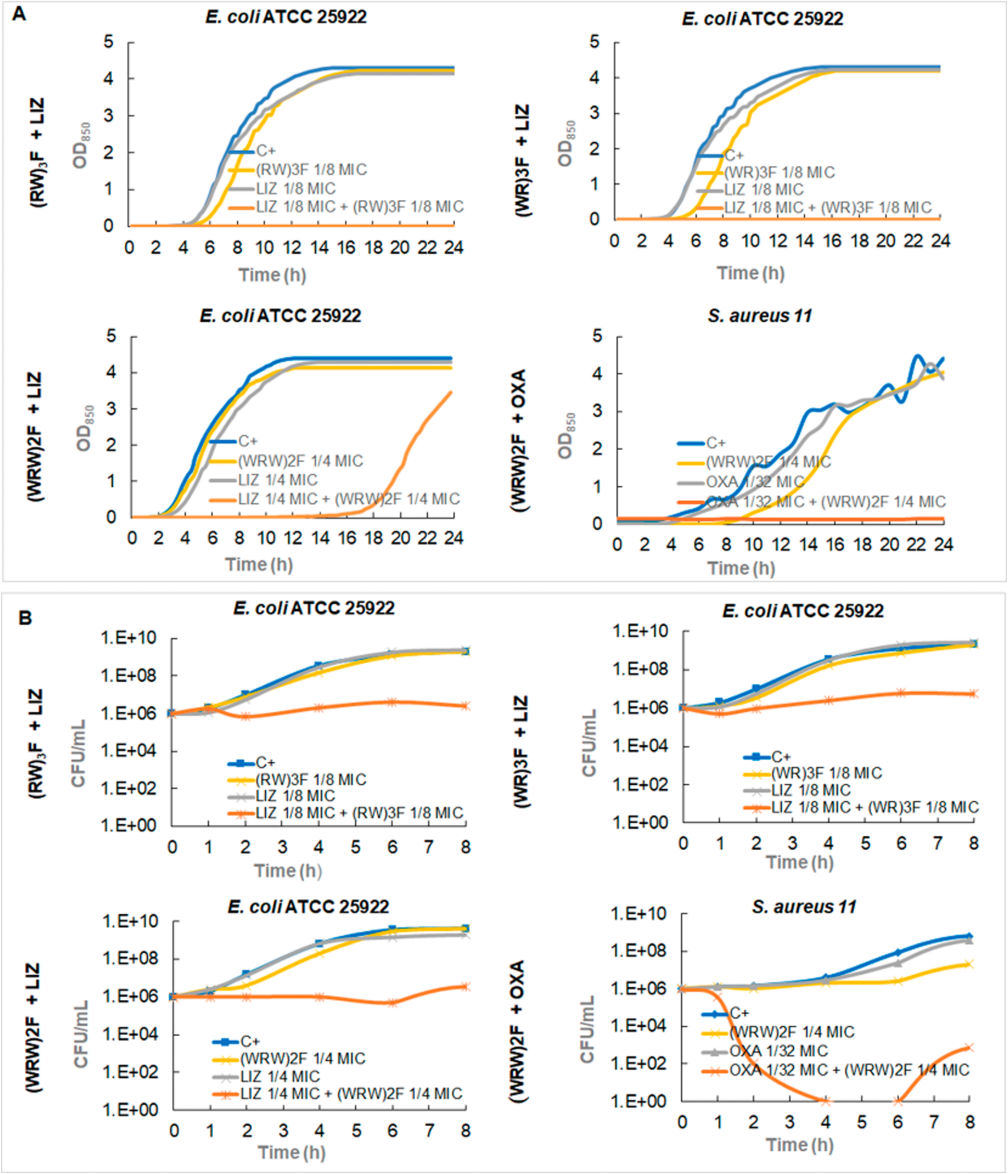
(A) Effect on growth curves of combinations displaying synergism (FICi ≤ 0.5) of RW peptides and conventional antimicrobials. LIZ (linezolid), OXA (oxacillin). (B)**.** Time-kill curves in the presence of synergistic combinations (FICi ≤ 0.5) of peptides and conventional antimicrobials. LIZ (linezolid), OXA (oxacillin).

Moreover, complete growth inhibition of *S. aureus* 11 was achieved when (WRW)_2_F and oxacillin were combined at concentrations of 1/4 MIC and 1/32 MIC, respectively (Figure 3A), further supporting the potential of (WRW)_2_F peptide to restore and enhance β-lactam efficacy against β-lactam-resistant strains.

Time-kill experiments revealed a bacteriostatic effect of the peptide–linezolid combinations against *E. coli*. At low concentrations (1/8 MIC) of the peptide, the antibiotic-peptide combination completely abolishes growth, whereas separately, none of the antimicrobials has an effect on growth. This finding suggests that the combination may be particularly effective in preventing bacterial proliferation, even in strains exhibiting reduced or no susceptibility to linezolid monotherapy (Figure 3B). Contrarily, in the case of the combination (WRW)_2_F and oxacillin against *S. aureus* 11, an evident bactericidal effect (of at least 6 h) is observed (Figure 3B), supporting the potential of (WRW)_2_F peptide to restore and enhance β-lactam efficacy against β-lactam-resistant strains.

The previous results are consistent with earlier studies, which show that, although linezolid is a well-known and potent antimicrobial compound primarily active against Gram-positive bacteria, its activity against Gram-negative bacteria (which are intrinsically resistant to linezolid) can be achieved when combined with antimicrobial peptides. In this sense, a previous study of our group found that the antimicrobial peptide colistin when combined with linezolid and rifampicin, was able to enhance their activity mostly due to colistin’s ability to destabilize bacterial outer membrane and suppress efflux mechanisms, which allow the penetration of linezolid and rifampicin.^23^ Additionally, a recent study by Huang et al. assessed the antibacterial efficacy of linezolid in combination with polymyxin derivatives (PBOP), suggesting that these combinations may be potential alternatives for combating *P. aeruginosa* infections.^24^ The effect of combinations herein studied on *P. aeruginosa* is less apparent than in *E. coli*. The superior synergistic activity observed between the peptides and linezolid in *E. coli* compared to *P. aeruginosa* can be attributed to the differences in outer membrane architecture and in efflux capacity. While *E. coli* expresses general trimeric porins, such as OmpF and OmpC, with low or even no substrate specificity, which facilitate the rapid influx of hydrophilic agents, this allows for sufficient intracellular accumulation to support synergistic interactions. In contrast, *P. aeruginosa* possesses a structurally distinct outer membrane with markedly lower permeability, primarily due to the presence of substrate-specific porins most monomeric with limited channel activity, and in addition a lipopolysaccharide-rich outer leaflet that adds more impermeability restricting drug entry. Additionally, *P. aeruginosa* constitutively expresses many potent efflux machinery complexes, notably MexAB-OprM, which are actively able to expel a broad range of antimicrobial agents.^25^ This combination of restricted permeability and active efflux hinders intracellular drug accumulation, thereby reducing the potential for synergistic effects.

The greater synergistic capacity of colistin and other polymyxins in *P. aeruginosa* is due to the catastrophic effects that polymyxin molecules exert on the structure of the outer membrane, effects that are visible under electron microscope. Additionally, findings on the combination of (WRW)­2F and oxacillin against *S. aureus* 11 align with previous studies reporting enhanced antibacterial activity when oxacillin is combined with membrane-active agents, such as nisin, particularly against MRSA strains. In addition, they indicate that certain antimicrobial peptides may contribute to restoring β-lactam susceptibility in resistant bacteria by compromising membrane integrity, thereby facilitating antibiotic uptake and offering a promising strategy for combination therapy.^26^

### Evaluation of the Efflux Pump Inhibition by Peptides

To explore the potential role of peptides as efflux pump inhibitors (EPIs), ciprofloxacin susceptibility was measured in the presence of known EPIs (CCCP for *E. coli*, PaβN for *P. aeruginosa* and reserpine for *S. aureus*), as well as (RW)_3_F, (WR)_3_F, (WRW)_2_F, against ciprofloxacin-resistant strains. Efflux pump overexpression is a well-established mechanism contributing to antimicrobial resistance, as it reduces the intracellular accumulation of antibiotics and other harmful compounds.^27,28^ Our results showed that the three peptides exhibited irrelevant effects (Figure S7). In previous works from our group on antimicrobial peptides,^29^ the ability of certain AMPs to mitigate the impact of efflux mechanisms and help overcome resistance associated with efflux pump overexpression was demonstrated. Supporting this, Armengol et al. demonstrated also that colistin not only increases membrane permeability and fluidity but also reduces efflux pump activity.^30^ Additionally, other studies have reported that AMPs such as Cecropin A can inhibit efflux pump activity in *E. coli*.^31^ In all cases, peptides acting on efflux pumps are much bigger than the peptides herein studied. When the uptake of acridine orange by the bacteria was measured in the presence of peptides, the susceptibility results were confirmed, since no differences in uptake levels were observed. Thus, it seems that none of the peptides act as EPI, which would mean that its mechanism of action is not related to reflux.

### Membrane Permeabilization Assay

To evaluate the membrane-permeabilising capacity of (RW)_3_F, (WR)_3_F and (WRW)_2_F, SYTOX green uptake was assessed by monitoring fluorescent changes at concentrations of 2× MIC, MIC and 1/2 MIC over 30 min for *E. coli* and *S. aureus*. SYTOX green is a membrane-impermeable fluorescent dye that penetrates damaged bacterial membranes and rapidly increases in fluorescence upon binding to nucleic acids.^32^

As shown in Figure 4, fluorescence intensity in *E. coli* ATCC 25922 increased in a concentration-dependent manner following peptide treatment. The highest fluorescence intensity was observed with (WRW)_2_F at 2× MIC, reaching 55,000 RFU (Relative Fluorescence Units) after 11 min and remaining stable throughout the 30 min assay. In comparison, (RW)_3_F and (WR)_3_F exhibited fluorescence intensities of approximately 40,000 RFU and 10,000 RFU, respectively, after 30 min. The lower increase in the fluorescence intensity found in the presence of (WR)_3_F suggests a lower membrane-permeabilization capacity compared to the other peptides.

**Figure 4:**
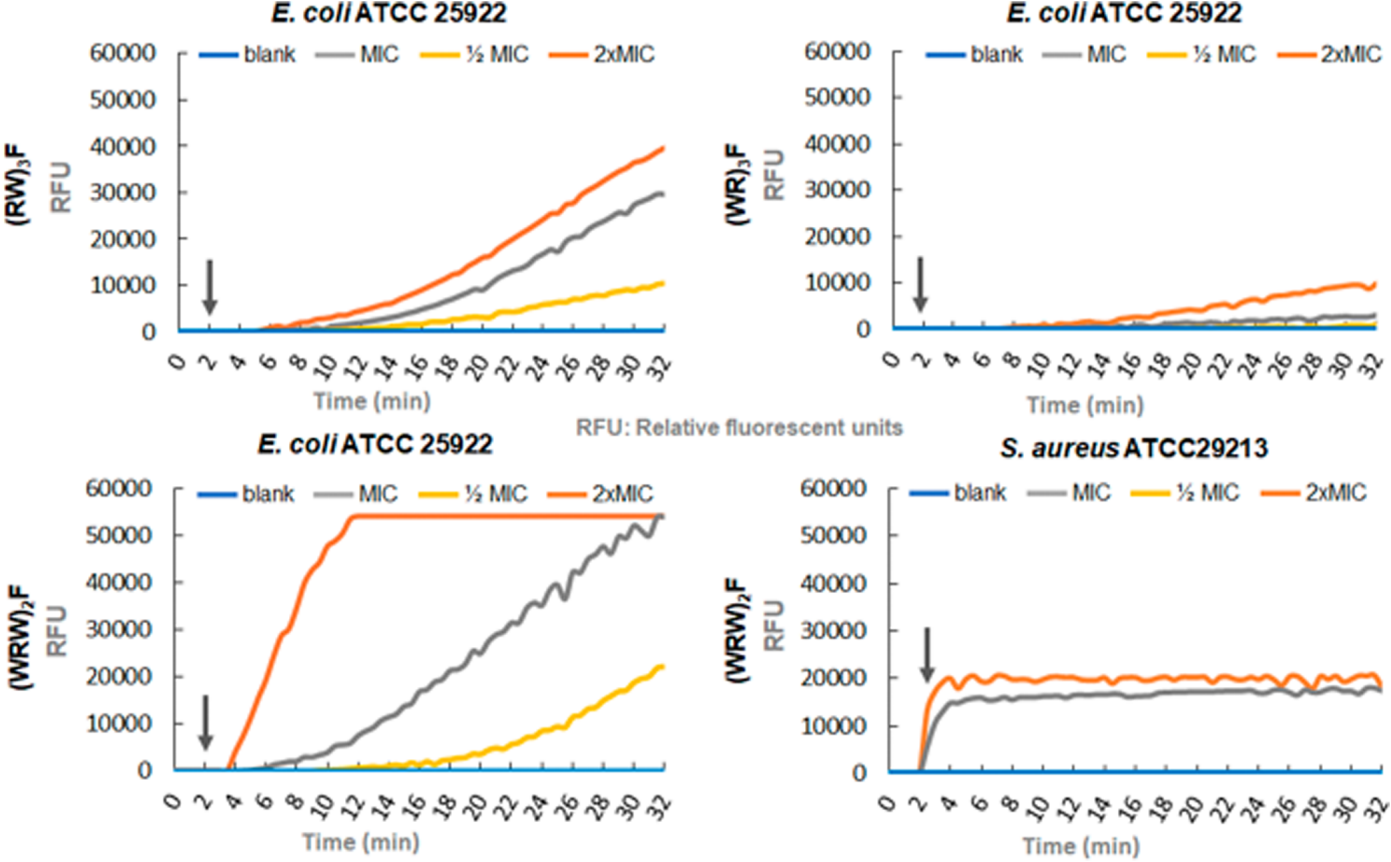
Changes in relative fluorescent units per minute in the presence of different concentrations of (RW)­3F, (WR)­3F, and (WRW)­2F. Black arrow: the addition of the peptide.

Additionally, the results demonstrate that (WRW)_2_F effectively and rapidly disrupted the membrane of *S. aureus*, with fluorescence reaching 20,000 RFU within 2 min of exposure. In contrast, the fluorescence in *E. coli* increased more gradually, indicating slower membrane permeabilization, similarly to the other peptides. This differential response suggests that (WRW)_2_F may target and disrupt the lipopolysaccharide (LPS) layer in Gram-negative bacteria. (Table 3). Fluorescence patterns in *E. coli* treated with (RW)_3_F, (WR)_3_F, and (WRW)_2_F also demonstrate that they exert rapid and direct membrane-disruptive effects.

Although (RW)_3_F and (WR)_3_F showed higher MICs against *E. coli* than (WRW)_2_F, all peptides affected permeability, which may explain their synergistic interaction with linezolid, as linezolid affects ribosomal function in both Gram-positive and Gram-negative bacteria but may not penetrate the outer membrane. Notably, fluorescence in both *E. coli* and *S. aureus* increased within 2–3 min of exposure to (WRW)_2_F, suggesting rapid and direct membrane damage. This is further supported by SYTOX Green uptake, AFM, and electron microscopy data.

Overall, (RW)_3_F, (WR)_3_F, and (WRW)_2_F exhibit rapid, dose-dependent membrane-permeabilizing activity in *E. coli* and *S. aureus*. This behavior is consistent with the capacity to alter bacterial membranes via electrostatic interactions and amphipathic structure, facilitating antibiotic entry and enhancing efficacy, particularly in Gram-negative bacteria such as *E. coli*.^33^ Our findings further support membrane permeabilization as a key factor in the synergistic potential of AMPs in combination therapies.

### Atomic Force Microscopy (AFM) and Transmission Electron Microscopy (TEM)

Amplitude and topography AFM images and Transmission electron micrographs obtained after treatment of *E. coli* ATCC 25922 with (RW)_3_F, (WR)_3_F and (WRW)_2_F at MIC and 1/2 MIC are shown in Figure 5, left panel. Untreated bacteria exhibited the typical rod-shaped with a well-structured membrane surface, whereas bacteria exposed to the three peptides displayed remarkable alterations. In spite of bacteria exposed to the peptide at MIC retained their characteristic rod-shaped morphology, membrane blebbing and surface wrinkling were evident. Occasionally, this was accompanied by visible cytoplasmic leakage, and a considerable accumulation of cellular debris was observed surrounding the bacterial cells. Treatment with the peptides at 1/2 MIC resulted in minor membrane surface corrugation and less pronounced membrane perturbation effects, such as vesiculation, except for (WRW)_2_F, which presents a drastic effect at both MIC and 1/2 MIC.

**Figure 5:**
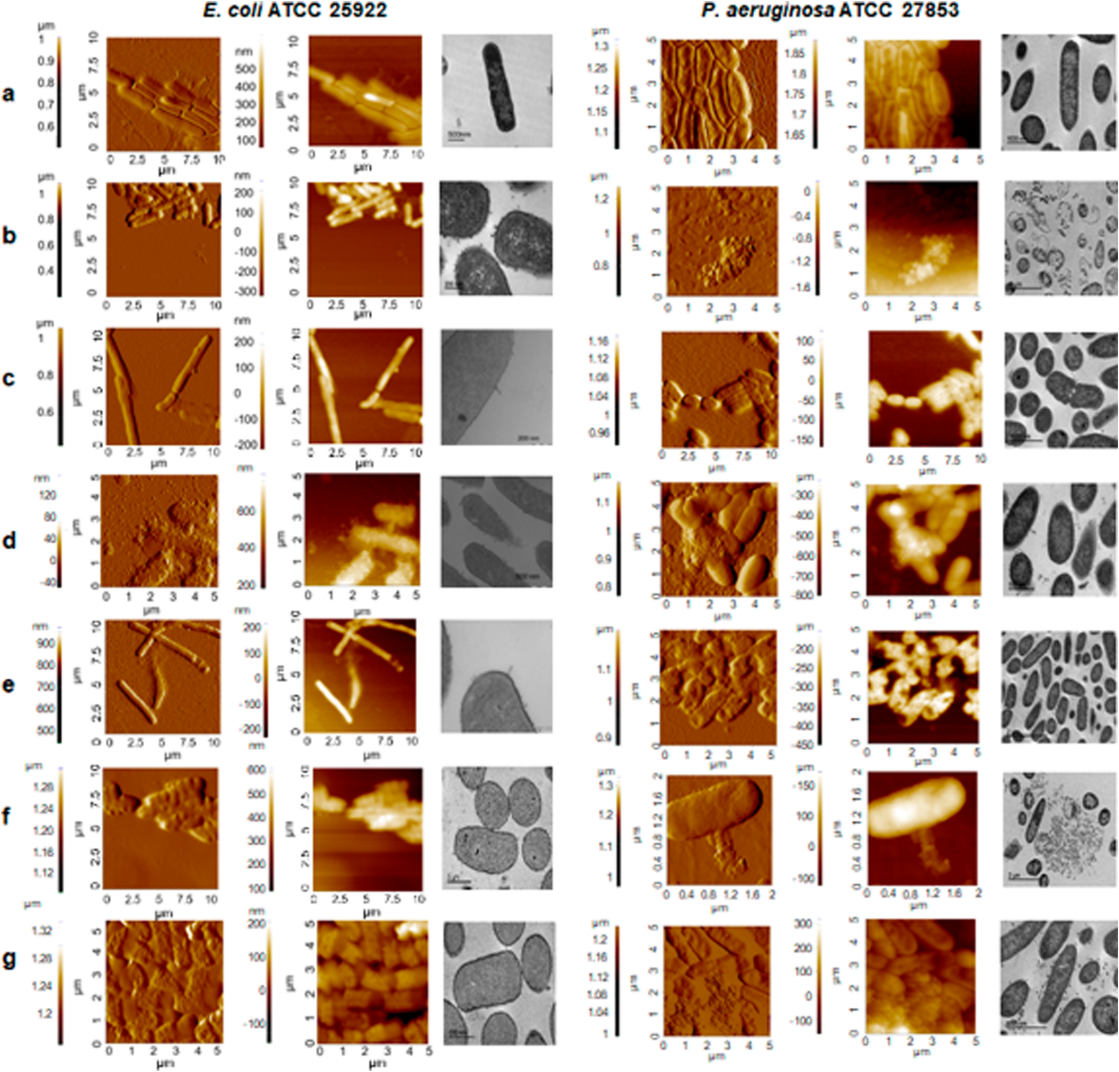
Amplitude, topography representative AFM and TEM images of *E. coli* ATCC 25922 left panel and *P. aeruginosa* ATCC 2785 right panel. (a) Untreated bacteria; (b) treated with (RW)­3F at MIC; (c) (RW)­3F at 1/2 MIC; (d) (WR)­3F at MIC; (e) (WR)­3F at 1/2 MIC; (f) (WRW)­2F at MIC; and (g) (WRW)­2F at 1/2 MIC.

Furthermore, elongation of bacterial cells was observed at subinhibitory concentrations of (RW)_3_F and (WR)_3_F, with minimal cell debris (Figure 5, left). Similar findings have been reported caused by other families of peptides unrelated with the ones herein studied such as pepR and BP100 at MIC levels that induced pronounced membrane alterations in *E. coli*, characterized by the form of corrugated surfaces and the formation of vesicle-like structures, accompanied by cytoplasmic leakage while at subinhibitory concentrations, the extent of damage was markedly reduced, with only minor perturbations to the bacterial envelope.^34^

Similar lesions in *E. coli*, including topographical irregularities, have been reported in cases involving magainin 2 amide and melittin. These alterations were attributed to peptide aggregation, incorporation of peptides into the LPS-containing outer membrane, release of LPS-enriched vesicles, or autolytic processes.^35^

In the case of *P. aeruginosa*, significant collapse in the bacterial envelope was observed, characterized by membrane disruption, vesicle formation and cytoplasmic leakage, particularly following treatment with (RW)_3_F and (WR)_3_F at the MIC (See Figure 5, right panel). In contrast, exposure to 1/2 MIC resulted in less pronounced envelope collapse and minor surface alterations. Similar to the effects observed in *E. coli*, substantial bacterial envelope perturbations and cytoplasmic release were evident at both MIC and 1/2 MIC for (WRW)_2_F. Strong alterations due to peptide-treatment have been reported by Viñas et al., who documented complete envelope disruption in the presence of the membrane-active colistin-inspired peptides.^36^ Here, the effects, although not as drastic may suggest that the peptides investigated may act in a similar way.

Finally, visualization of the effects of peptide action on *S. aureus* allows us to observe significant changes in the bacteria treated with the peptide (WRW)_2_F at the MIC. Most bacteria were completely lysed, resulting in the leakage of cytoplasmic contents. At 1/2 MIC, some bacterial cells maintained their characteristic spherical morphology, while others exhibited a loss of bacterial structure, with the cell wall being completely damaged, which ultimately led to bacterial lysis (Figure 6). A previous study by Domingues et al. found similar effects on the bacterial cell wall, characterized by a loss of structure in the presence of the peptide rBPI2. As the negatively charged lipoteichoic acid (LTA) of *S. aureus* has similar physicochemical properties to the LPS of Gram-negative bacteria, Domingues et al. hypothesized that alterations to the cell wall and cytoplasmic membrane, including loss of cellular content, could be a consequence of the interaction of the peptide with LTA.^37^ This was also demonstrated in colistin-derived peptides acting on Gram-positives, also through the action on teichoic acids.^38^

**Figure 6:**
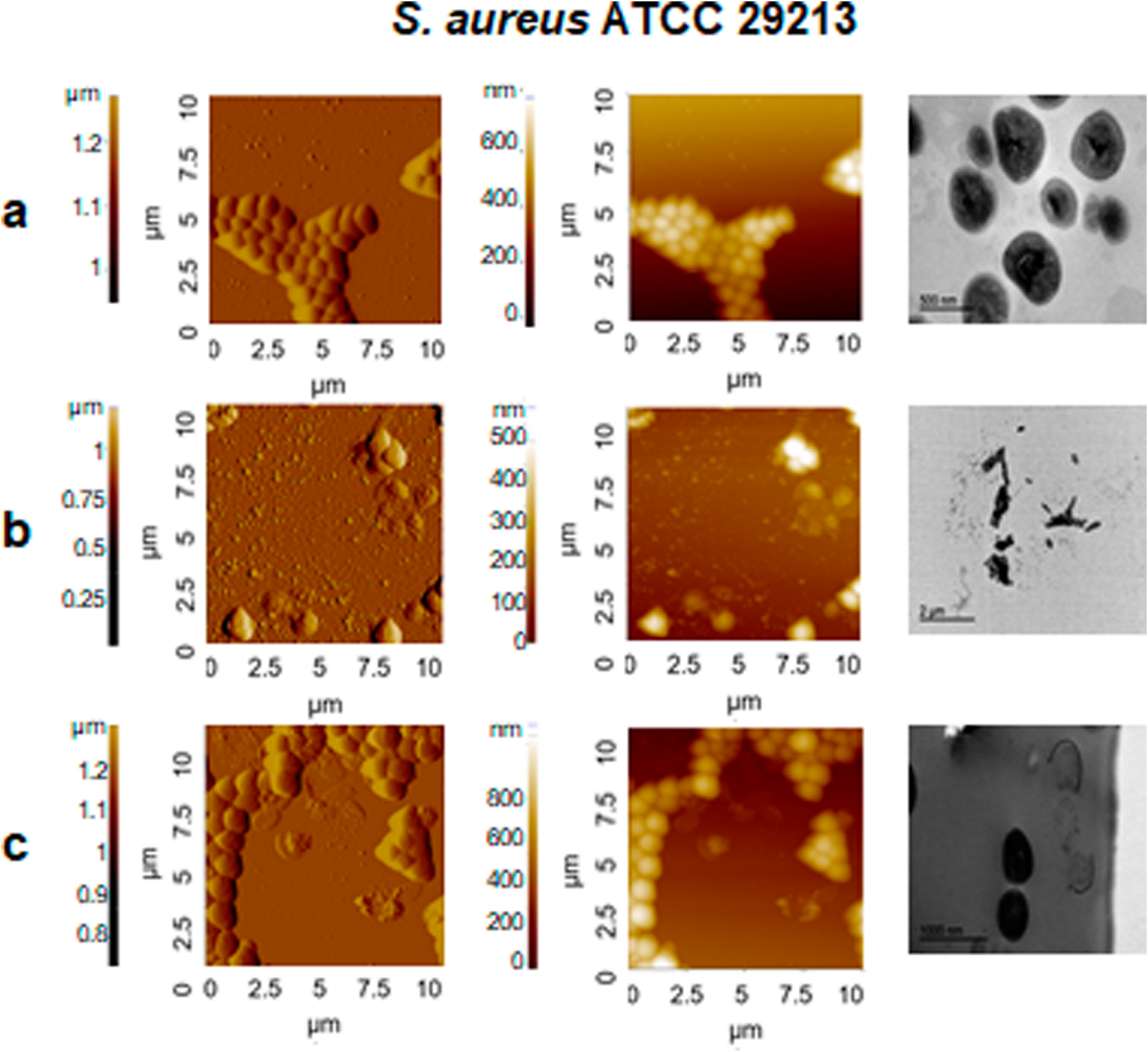
Amplitude, topography and TEM images of *S. aureus* ATCC 29213. (a) Untreated bacteria; (b) treated with (WRW)­2F at MIC; and (c) treated with (WRW)­2F at 1/2 MIC.

TEM visualizations confirmed the results under AFM since treated bacteria displayed altered morphology, including membrane disruption and bleb formation in Gram negatives (Figures 5 and 6). A recent study by Song et al. reported comparable membrane alterations in *E. coli* following exposure to the short cationic peptide Trp-rich GW4A. These changes were attributed to compromised membrane integrity induced by the peptides, leading to cytoplasmic leakage and subsequent cell death.^39^

As with the observations for *E. coli*, peptide-treated *P. aeruginosa* showed bleb formation. Treatment at the MIC by (RW)_3_F and (WRW)_2_F resulted in increased bacterial leakage and the release of cytoplasmic content, accompanied by substantial amounts of cell debris. This suggests that these peptides interact with cell membranes, likely causing cell death or alterations that contribute to the synergistic effects described. At 1/2 MIC, bleb formation was mainly observed after treatment with (WRW)_2_F, while release of cytoplasmic content was absent. A recent study by Fahmy et al. showed similar effects, inducing membrane rupture and leakage of cytoplasmic content with extracellular cell debris, when *P. aeruginosa* was treated with the AMP His-Hill_BB_C10074 from the attacin family.^40^

For *S. aureus*, treatment at the MIC concentration by (WRW)_2_F resulted in extensive structural damage, including clear signs of membrane collapse and marked accumulation of cellular debris. At 1/2 MIC, some bacteria maintained their native morphology, while others showed complete rupture and leakage of intracellular contents. These findings align with the morphological alterations observed by AFM.

A similar effect was reported by Zhu et al., who observed strong membrane disruption and leakage of intracellular contents in *S. aureus* following treatment with the cationic peptide PRW4.^41^

### Toxicity

Since one of the main obstacles to the clinical therapeutic use of AMPs is their possible toxic effects on mammalian cells,^42,43^ we evaluated the toxicity of the peptides in two mammalian cell lines, N-929 (mouse fibroblast) and HepG2 (human liver cancer), as well as in *Caenorhabditis elegans* to assess in vivo safety.^44^

Among the peptides tested, (WRW)_2_F exhibited the most favorable safety profile, with high IC50 values in both cell lines (232.70 μg/mL in N-929 and 242.62 μg/mL in HepG2), indicating low cytotoxicity. In contrast, (WR)_3_F showed the highest toxicity, particularly against hepatic cells (IC50 of 62.10 μg/mL in HepG2 and 113.10 μg/mL in N-929). (RW)_3_F displayed intermediate toxicity, with IC50 values of 114.44 μg/mL in HepG2 and 161.64 μg/mL in N-929 (Figure 7A).

**Figure 7:**
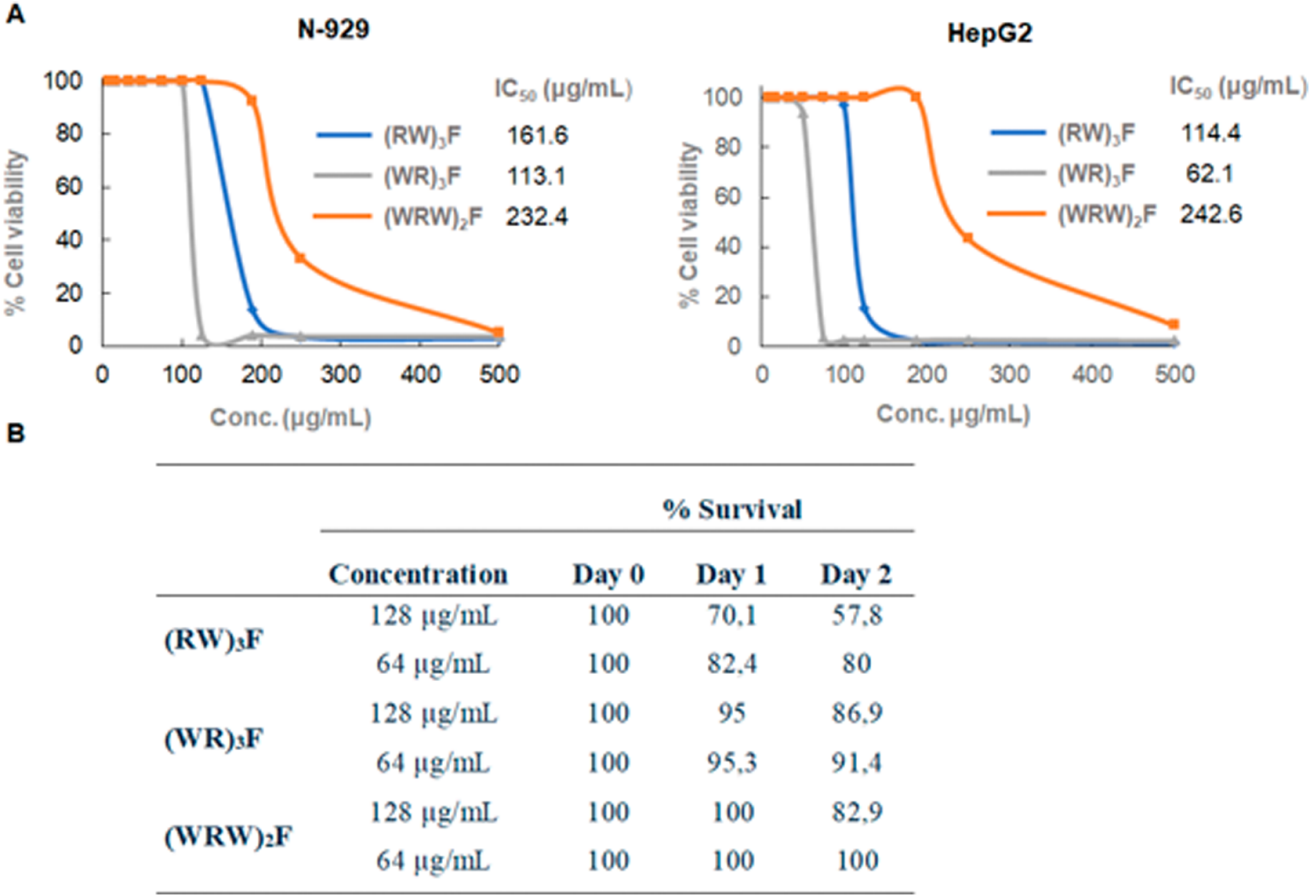
(A) Percentage of cell viability of N-929 (fibroblasts) and HepG2 (hepatocytes) in the presence of a wide range of concentrations of the peptides (RW)­3F, (WR)­3F, and (WRW)­2F (500–8 μg/mL). (B) Survival of *C. elegans* after exposure to RW peptides.

However, as mentioned earlier, the hypothetical use of this peptide family would lie in its activity as sensitizers to conventional antibiotics; this activity occurs at concentrations of 4 μg/mL or lower, which places it far from the established toxicity levels, meaning that at these concentrations, they could be considered completely nontoxic.

The *in vivo* safety profile of the peptides was further assessed using *C. elegans* as a model organism, with survival monitored over a 48 h period at two concentrations (128 μg/mL and 64 μg/mL). Consistent with the in vitro results, (WRW)_2_F maintained 100% survival at 64 μg/mL and showed only a modest reduction to 82.9% at 128 μg/mL. (WR)_3_F also exhibited relatively low toxicity, with survival rates of 91.4 and 86.9% at 64 μg/mL and 128 μg/mL, respectively. Although (RW)_3_F showed a more pronounced effect on survival, the data remain consistent with its intermediate cytotoxicity in mammalian cells. Overall, these findings highlight the promising therapeutic potential of (WRW)_2_F, which combines antimicrobial efficacy with excellent biocompatibility across both in vitr*o* and in vivo models.

These results are consistent with previous studies on RW-based peptides, which have shown that increasing chain length enhances antimicrobial activity but also raises cytotoxicity. Liu et al. demonstrated that (RW)_3_ represents an optimal balance between synthesis efficiency and biological activity, with strong interaction with negatively charged membranes.^11^ More recently, Sunda-Meya et al. introduced proline-modified RW peptides that significantly reduced cytotoxicity while maintaining broad-spectrum antimicrobial efficacy.^18^ Our findings reinforce these observations and highlight the value of rational peptide design.

The incorporation of structural modifications in (WRW)_2_F led to excellent tolerability in *C. elegans* (Figure 7B), supporting its potential as a safe and effective candidate for therapeutic development. In any case, the use of the three peptides at the concentrations necessary to resensitize the bacteria to the antibiotics to which they had become resistant remains well below the detected toxicity limits

## Conclusions

Short Arg/Trp-rich peptides have been evaluated as antimicrobial candidates. Among the sequences tested, (WRW)_2_F surpassed (RW)_3_F and (WR)_3_F in both Gram-positive and Gram-negative bacteria, displaying the strongest antimicrobial activity. Its enhanced efficacy appears linked to an optimal balance of hydrophobicity and net positive charge. While exploring the possible mode/s of action, it has been established that none of the peptides significantly affected efflux-related ciprofloxacin susceptibility or acridine-orange accumulation, thus their activity does not rely on interfering with efflux systems. However, techniques such as SYTOX Green uptake, AFM, and TEM revealed a rapid membrane permeabilization occurring in a dose-dependent way, with the (WRW)_2_F analogue being the one causing the most pronounced structural damage. This disruption facilitates antimicrobial activity and supports the synergistic interactions found with certain antibiotics, since all three peptides displayed synergy with linezolid against *E. coli*, while (WRW)_2_F restored oxacillin susceptibility in MRSA. On the other hand, cytotoxicity assays and *C. elegans* survival studies demonstrate that (WRW)_2_F is substantially less toxic than the other peptides, with effective concentrations far below toxicity thresholds.

Then, our study reinforces the promise of RW-based peptides as versatile scaffolds for next-generation antimicrobial agents, and highlights (WRW)­2F as a particularly compelling candidate due to its favorable safety profile and more potent activity.

From our perspective, two aspects of the work are worth highlighting. First, the peptides studied are short, easy to synthesize, and therefore would be economically affordable. Second, although the compounds do not possess extraordinary antibacterial activity per se, their ability to resensitize bacteria resistant to conventional antibiotics underscores their potential value in combination therapies.

## Experimental Section

### General

All solvents and reagents used for the synthesis were bought from commercial suppliers and were used further without any purification unless otherwise indicated. Fmoc amino acids and Fmoc Rink amide PS-resin (0.74 mmol/g) were purchased from Iris Biotech GmbH (Marktredwitz, Germany). DIC and OxymaPure were gifts from Luxembourg Bio-Technologies. LCMS was performed on an Ultimate 3000 using an AerisTM 3.6 μm wide pore column from Phenomenex C18 (4.6 mm × 150) (system 2). Buffer A: 0.1% formic acid in H2O; buffer B: 0.1% formic acid in CH3CN, flow 1.0 mL/min, UV detection 220 nm.

*Escherichia coli* ATCC 25922, *Pseudomonas aeruginosa* ATCC 27853, *Staphylococcus aureus* ATCC 29213, were used as control strains. The clinical isolates: *E. coli* 7987, *E. coli* 208691, *P. aeruginosa* 230531, *P. aeruginosa* 666 SJD, *S. aureus* 08, *S. aureus* 11, and *S. aureus* 19, were also included. All the strains were obtained from our own collection.

Tryptone soy agar (TSA) was purchased from Sharlau (Barcelona, Spain), and Mueller Hinton II broth cation-adjusted (MHBCA) from Becton Dickinson Diagnostic Systems, Inc. (Sparks, MD, USA). Efflux pump inhibitors, including Carbonyl Cyanide m-Chlorophenylhydrazone (CCCP), Phe-Arg β-naphthylamide dihydrochloride (PaβN), and reserpine, as well as antibiotics such as imipenem monohydrate (IMI), linezolid (LIZ), tetracycline (TET), and oxacillin (OXA), were obtained from Sigma-Aldrich Chemicals (Madrid, Spain). The Sytox Green solution was acquired from Invitrogen (SYTOX Green S7020, Eugene, OR, USA).

### Peptide Synthesis

All peptides were synthesized on a 0.1 mmol scale using standard Fmoc/tBu solid-phase peptide synthesis (SPPS) protocols with DIC and OxymaPure as coupling reagents on Fmoc-Rink-Amide AM resin (loading = 0.64 mmol/g). Syntheses were performed manually at room temperature in polypropylene syringes equipped with polypropylene frits. Fmoc deprotection was achieved by treating the resin with 20% piperidine in DMF for 1 min, followed by a second treatment for 7 min. Coupling reactions were carried out using a 3-fold molar excess of Fmoc-protected amino acid, DIC, and OxymaPure (1:1:1 ratio) in DMF for 45 min. Upon completion of chain elongation, the peptidyl resin was washed with methanol and dried under vacuum. Peptides were cleaved from the resin with TFA/H_2_O/TIS (95:2.5:2.5, v/v/v) for 1 h at room temperature. The crude peptides were precipitated with chilled diethyl ether, centrifuged, and the supernatant discarded. The precipitate was washed twice with cold diethyl ether, centrifuged again, and dried overnight under vacuum in a desiccator. Dried peptides were dissolved in 10% acetic acid and analyzed by RP-HPLC and LC–MS. Final purification was performed using semipreparative HPLC to achieve a purity of at least 95%.

### Evaluation of Antimicrobial Activity

#### Minimal Inhibitory Concentration (MIC)

The antimicrobial activity of (RW)_3_F, (WR)_3_F, (RWR)_2_F, (WRW)_2_F, (RW)_3_Fc and (WR)_3_Fc was initially evaluated by determining the minimum inhibitory concentration (MIC) using the reference broth microdilution method in 96-well microtiter plates, as recommended by CLSI.^45^ Briefly, bacteria were grown overnight in MHBCA and then adjusted to an OD_625 nm_ of 0.08–0.1. Five microliters of each bacterial suspension were added to wells previously filled with MHBCA, and the peptides were serially diluted from 64 to 0.125 μg/mL. Plates were incubated at 37 °C for 24 h. MIC values were determined by macroscopic observation of the turbidity in the wells. All experiments were performed in triplicate. Based on the MIC values, certain strains were excluded from further experiments.

#### Growth Curves

The effect of the peptides on bacterial growth was analyzed using the following protocol. Briefly, the peptides were added at inhibitory (MIC) and subinhibitory (1/2 MIC and 1/4 MIC) concentrations to bacterial cultures in MHBCA and incubated in real-time reverse spin bioreactors RTS-1 (Biosan SIA, Riga, Latvia) at 37 °C and 2000 rpm for 24 h. Growth was measured noninvasively at an optical density of 850 nm every 15 min for 24 h. All measurements were performed in triplicate.

#### Time-Kill Curves

The death kinetics caused by active peptides were explored performing a time-kill assay. (RW)_3_F, (WR)_3_F and (WRW)_2_F at different concentrations (MIC, 1/2 MIC and 1/4 MIC) were added to the bacterial cultures and incubated for 24 h at 37 °C, with shaking at 200 rpm. Samples were aseptically obtained at 0, 1, 2, 4, 6, and 8 h, serially diluted in Ringer 1/4 and plated on TSA for colony counting. Plates were incubated for 24 h at 37 °C. The response of the strains to the peptides was determined based on a logarithmic decrease in viable bacteria. Time-kill assays were performed in triplicate.

#### Interactions

A checkerboard test was performed, as previously described by Chen et al.,^46^ to determine the fractional inhibitory concentrations (FICs) of (RW)_3_F, (WR)_3_F and (WRW)_2_F in combination with conventional antibiotics against *E. coli* ATCC 25922, *P. aeruginosa* ATCC 27853, and a clinical isolate *S. aureus* 11­(MRSA). In brief, each well in a 96-well plate was inoculated with 100 μL of a bacterial inoculum of 5 × 10^5^ CFU/mL and the different combined concentrations of the two antimicrobials. The plates were incubated at 37 °C for 24 h. The FIC was calculated after identifying the first well in each row without growth (MIC), according to the following formula: FIC of drug A (FIC A) = (MIC of drug A in combination)/(MIC of A); FIC of drug B (FIC B) = (MIC of drug B in combination)/(MIC of B). The FIC index (FICi) values were calculated by adding the FIC A to the FIC B and interpreted as follows: FICi ≤ 0.5, synergistic; FICi between 0.5 and 4, indifferent; FICi ≥ 4, antagonistic.^47^ The effect of the combined concentrations used to calculate the FICi on bacterial growth was then investigated by plotting growth curves.

#### Evaluation of the Efflux Pump Inhibition of Peptides

Efflux pump inhibition capacity of peptides was explored in ciprofloxacin-resistant bacteria as previously described by Baron et al. with some modifications.^48^ Briefly, a 96-microtiter plate was filled with MHBCA with ciprofloxacin concentration range of 512–0.5 μg/mL. Then, the bacterial inoculum in logarithmic phase was added. Two tests were performed in parallel: one without adding the efflux pump inhibitors (EPIs) or peptides to each MHBCA wells and the other by adding the known EPIs (CCCP at 10 μg/mL for *E. coli* 208691; PaβN at 20 μg/mL for *P. aeruginosa* 666 SJD and Reserpine at 25 μg/mL for *S. aureus* 19) or subinhibitory concentrations of peptides. A growth control well containing the EPI or peptide in MHBCA was also added for each strain, to check the absence of effect alone. The resulting MIC fold changes after the addition of known EPIs and peptides was calculated as the ratio of the known EPI or peptide-free ciprofloxacin’s MIC level to that of the EPI or peptide-added ciprofloxacin.

#### Membrane Permeability Assay

The ability of the peptides (RW)_3_F, (WR)_3_F and (WRW)_2_F to permeabilise bacterial membranes was evaluated by assessing the uptake of SYTOX Green by bacterial cells, following the protocol described by Kim et al. with minor modifications.^49^ In brief, bacterial cultures were adjusted to an OD600 of 0.4 (approximately 2 × 10^8^ CFU/mL), then centrifuged and resuspended in an equal volume of PBS. SYTOX Green was then added to each bacterial suspension at a final concentration of 5 μM. The samples were then incubated at 37 °C for 30 min, in the dark. Then, 50 μL of the bacterial/SYTOX Green mixture was transferred to each well of a black, clear-bottom 96-well microplate (Thermo Fisher Scientific, Rochester, New Yorkbrand). Fluorescence was recorded every 30 s for 30 min at room temperature using a FLUOstar OPTIMA microplate reader (BMG Labtech, Ortenberg, Germany), with excitation and emission wavelengths set at 485 and 525 nm, respectively. Peptides were added after the initial 2 min of measurement, diluted in 50 μL of phosphate buffered saline (PBS) to achieve final concentrations corresponding to 2xMIC, MIC and 1/2 MIC. Untreated bacteria was used as control.

#### Atomic Force Microscopy (AFM)

AFM was used to visualize the morphological alterations on the surfaces of untreated and treated *E. coli* ATCC 25922, *P. aeruginosa* ATCC 27853 and *S. aureus* ATCC 29213 following exposures to (RW)_3_F, (WR)_3_F and (WRW)_2_F at MIC and 1/2 MIC. Briefly, bacterial cultures at a concentration of 10^8^ CFU/mL were incubated with different concentrations of peptides. Subsequently, the bacteria were collected by centrifugation at 5000 rpm for 3 min and the resulting pellet was resuspended in sterile water. A 10 μL volume was applied to a Thermanox surface, dried at room temperature and then, imaged in air by using an atomic force microscope XE-70 (Park Systems, Korea) in noncontact mode with an ACTA 10 M cantilever.^35,50^

#### Transmission Electron Microscopy (TEM)

TEM was used to analyze the phenotypic alterations of the treated and untreated *E. coli* ATCC 25922, *P. aeruginosa* ATCC 27853 and *S. aureus* ATCC 29213 with MIC and 1/2 MIC of (RW)_3_F, (WR)_3_F and (WRW)_2_F. Time-kill curves provided us information to decide on exposure times. Brief, 10^8^ CFU/mL of starting inoculum were incubated with the different concentrations of peptides. The cultures were then centrifuged at 5000 rpm for 5 min and the pellet was fixed with 2.5% glutaraldehyde and 2% paraformaldehyde in 0.1 M phosphate buffer for 30 min at room temperature. The fixed samples were centrifuged at 5000 rpm for 5 min and the pellet was resuspended in the fixing solution and stored at 4 °C. Bacteria were postfixed with osmium tetroxide, dehydrated with acetone, embedded in resin and sectioned with an ultramicrotome. Ultrathin sections (50–70 nm) were stained with 2% uranyl acetate for 10 min, a lead staining solution for 5 min and finally analyzed on a JEOL 1010 the transmission electron microscope with a CCD Orius digital camera (Gatan).

#### Toxicity

Cell viability in the presence of (RW)_3_F, (WR)_3_F and (WRW)_2_F was evaluated using the fluorometric resazurin assay. Resazurin is a redox-sensitive dye which is reduced by viable cells to resorufin, a fluorescence compound. Human hepatocarcinoma cells (HepG2) and fibroblasts (N-929) were used in the drug toxicity assays. Viable HepG2 and N-929 cells (20,000 cells/well) were seeded into a 96-well microtiter plate (200 μL) and allowed to attach overnight. The cells were exposed to ranged concentrations of the peptides from 500 to 8 μg/mL for 24 h. After removal of the treatment medium, resazurin was added to each well at a final concentration of 70 μM. After a 24 h incubation at 37 °C, the fluorescence was measured at an excitation wavelength of 530 nm and an emission wavelength of 590 nm using a FLUOstar OPTIMA fluorescence microplate reader (BMG Labtech, Ortenberg, Germany).

The results were reported as percentage of cell viability (Absorbance of treated cells/Absorbance of control cells) × 100) versus the concentration range tested. GraphPad Prism (V5) (La Jolla, CA, USA) was used to obtain the half-maximum inhibitory concentration (IC_50_) for each compound.

The toxicity assay with the nematode *C. elegans* was used to evaluate nematode survival rates in the presence of different concentrations of peptides. Larval synchronization was performed following the protocol described in The WormBook (https://www.wormbook.org/toc_complete.html). Briefly, unseeded nematode growth medium (NGM) plates were used to isolate embryos hatch and arrest at the L1 stage in the absence of food. Embryos were isolated using an alkaline hypochlorite solution that dissolves gravid hermaphrodites without damaging their embryos. After incubation, L1 larvae were transferred to plates of nematode culture medium (NGM) with *E. coli* OP50 for growth to the desired stage. L1 larvae were grown until L4 stage on agar plates seeded with an *E. coli* bacterial lawn, and the survival assay was performed at room temperature by inoculating between 15 and 20 L4 nematodes per plate on NGM medium after nematode exposure to RW peptides (*E. coli* OP50 seeded with untreated larvae’s was used as a control). Nematode survival was registered by observation of the seeded plates at the binocular loupe (Carton Optics, Japan), assessing whether the larvae move or not. Counting was performed at times 0, 24, and 48 h. All experiments were performed by triplicate.^51^

## Supplementary Material



## Notes

HPLC traces and ESI-MS of the peptides (PDF)
