# Supplementary material for: Short Antimicrobial Peptides Based on Arginine and Tryptophan: Agents with Potential in Combating Resistant Pathogens
Source: ACS Omega. 2026 Mar 13;11(11):18010–23. doi: 10.1021/acsomega.5c12724 (PMC13019373; doi:10.1021/acsomega.5c12724)

Short antimicrobial peptides based on Arginine and Tryptophan, agents with potential in combating resistant pathogens

Eric Fernández de la Cruz[1,†], Jessica T. Mhlongo[2,†], Ashish Kumar[2] Fernando Albericio[4,5], Miguel Viñas[1], Paula Espinal[1] , Ester Fusté[1,3]* and Beatriz G. de la Torre [2]*

* Correspondence: esterfustedominguez@ub.edu (E.F.); garciadelatorreb@ukzn.ac.za (B.G.d.l.T.)

† These authors contributed equally to this work.

UV-HPLC traces and ESI of the purified peptides synthesised

Figure S1: A) HPLC trace of (RW)$_3$F. Gradient 5 to 95% B into A in 15 min UV detection at 220nm. B) ESI MS, inserted the molecular structure.

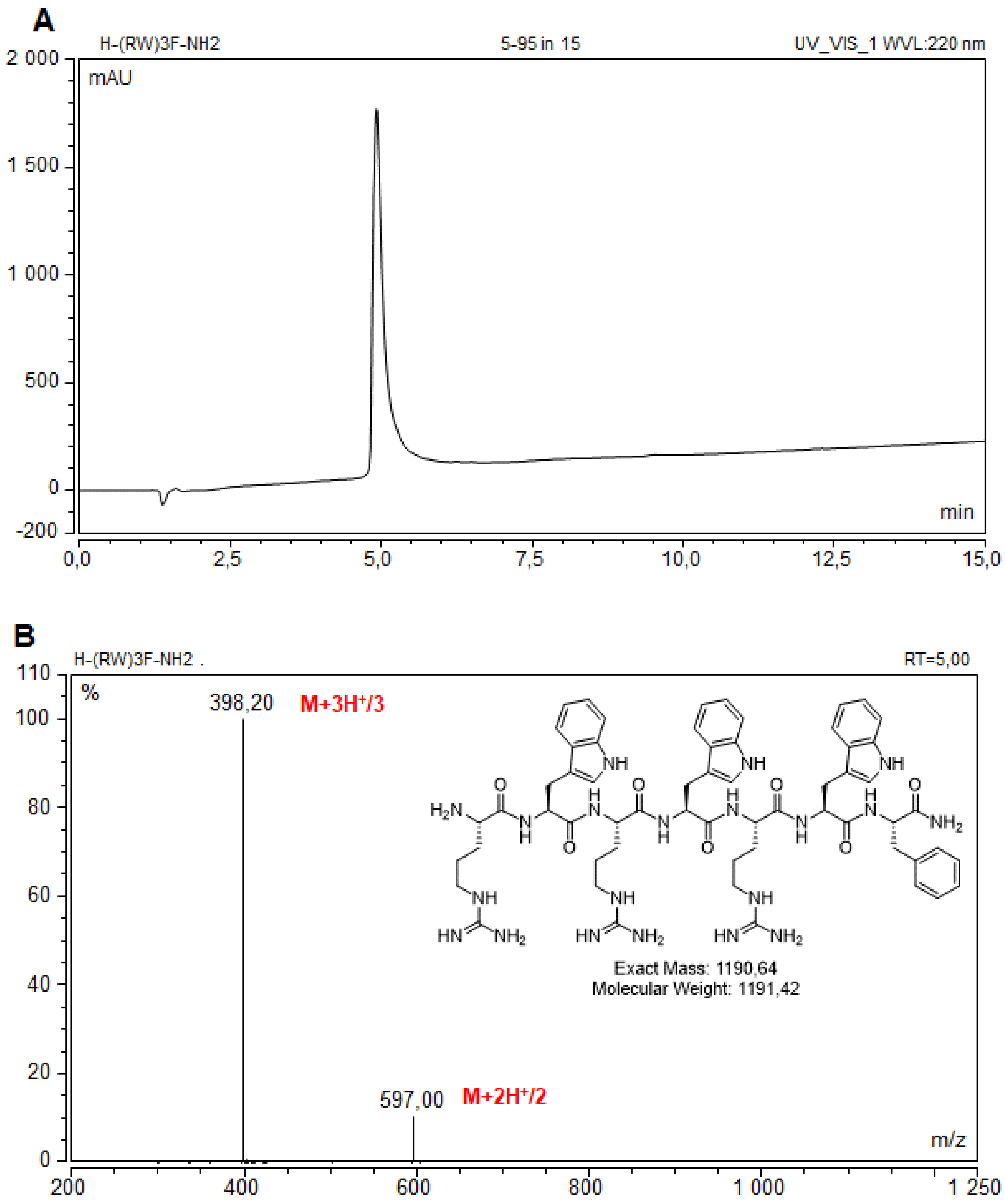

Figure S2: A) HPLC trace of $(WR)_3F$. Gradient 5 to 60 % B into A in 15 min UV detection at 220nm. B) ESI MS, inserted the molecular structure.

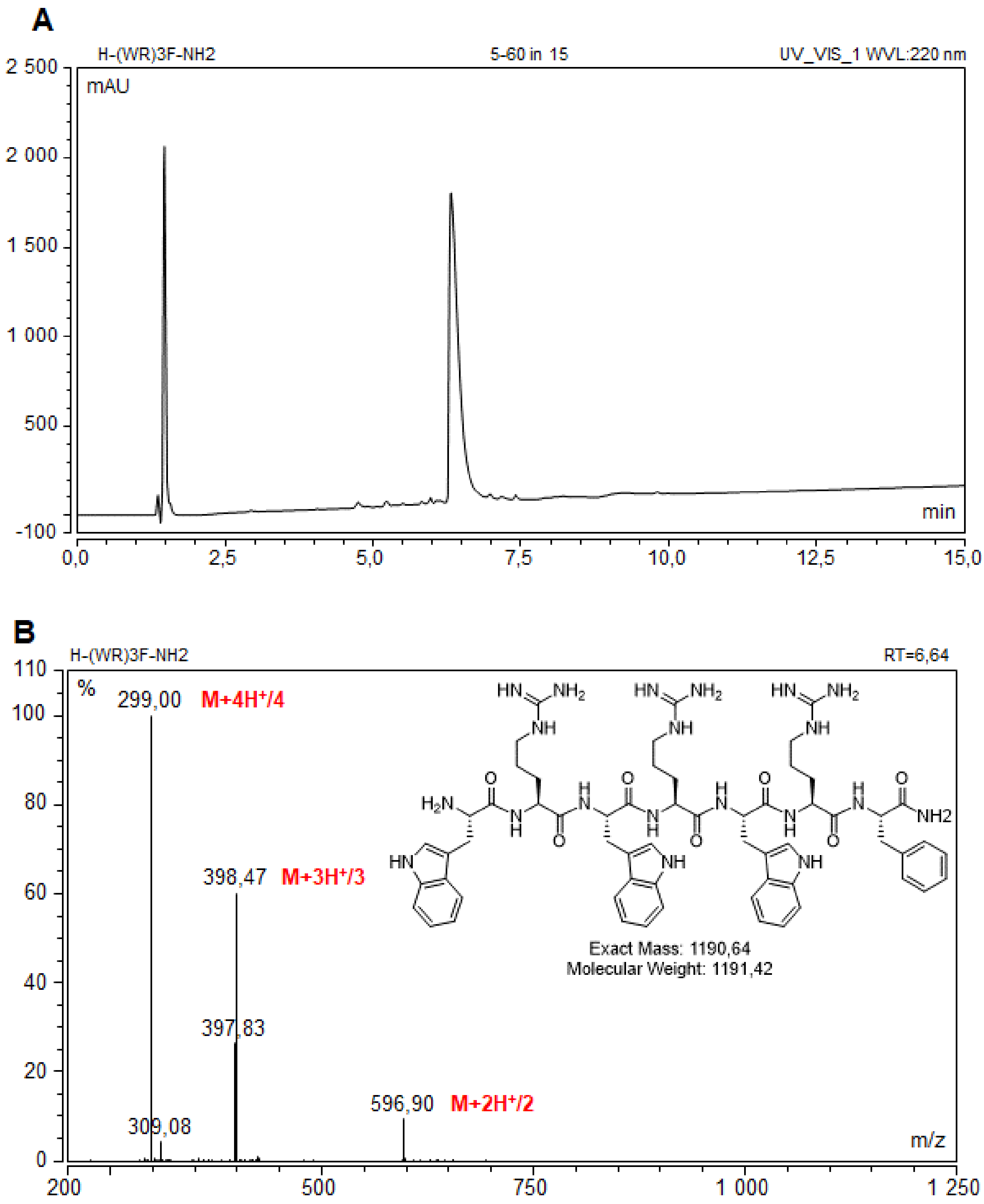

Figure S3: A) HPLC trace of $(RWR)_2F$. Gradient 5 to 60 % B into A in 15 min UV detection at 220nm. B) ESI MS, inserted the molecular structure.

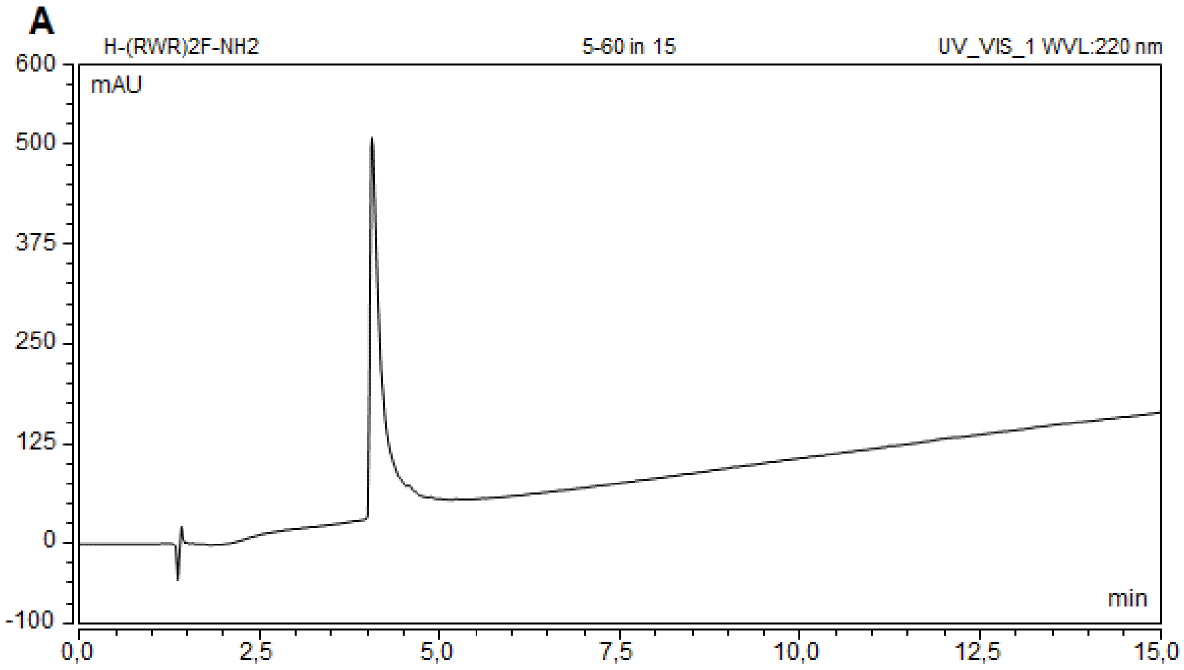

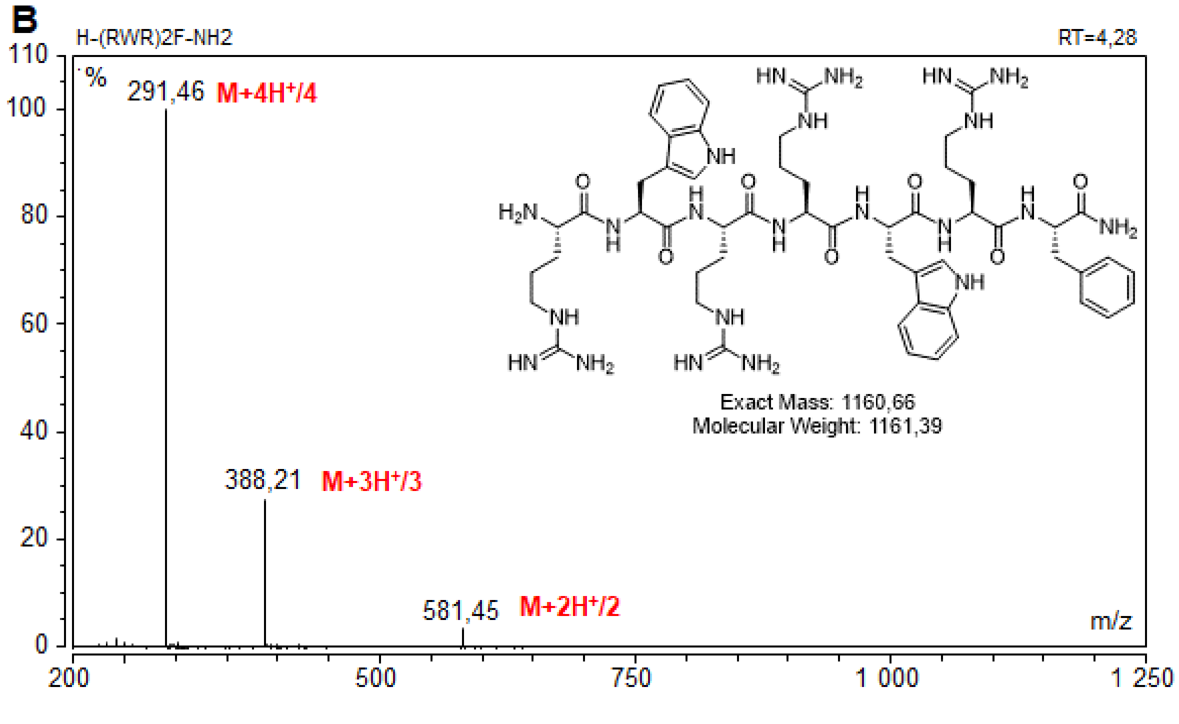

Figure S4: A) HPLC trace of (WRW)$_2$F. Gradient 10 to 70 % B into A in 15 min UV detection at 220nm. B) ESI MS, inserted the molecular structure.

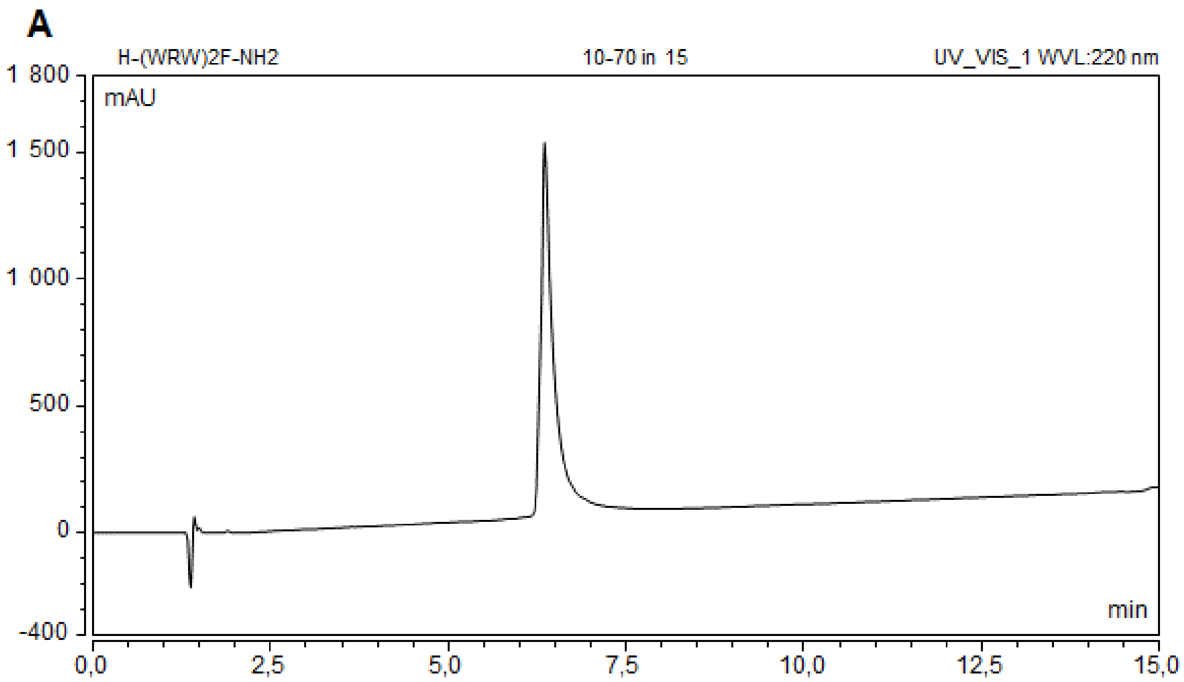

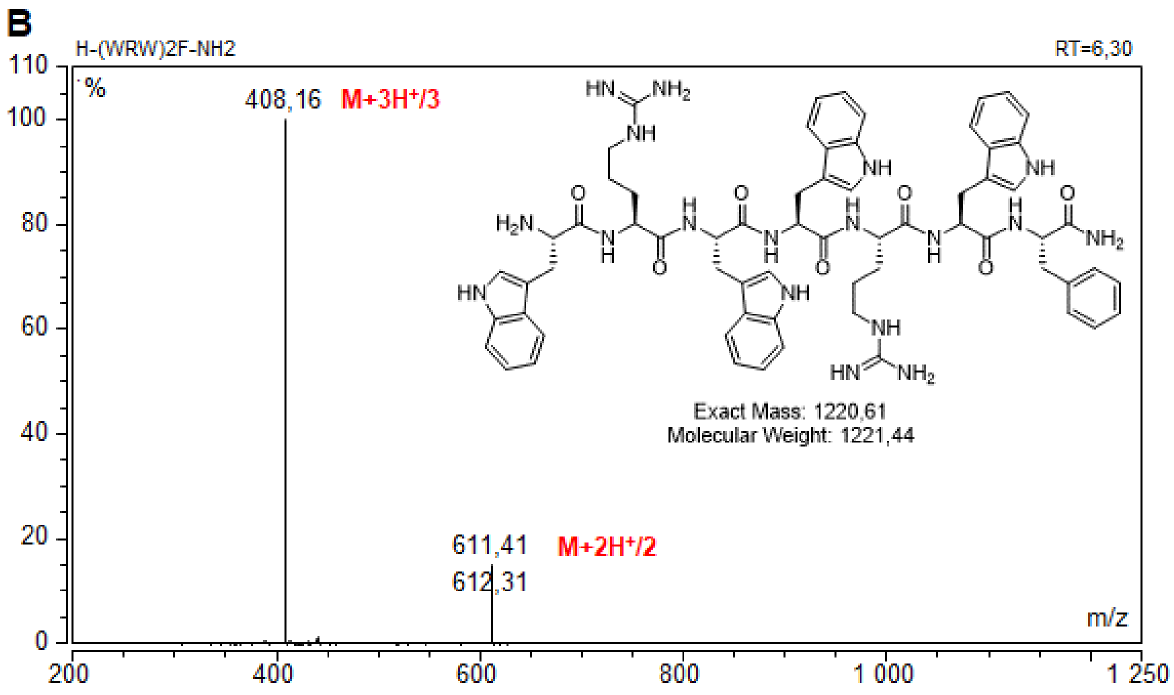

Figure S5: A) HPLC trace of (RW)$_3$Fc. Gradient 5 to 95% B into A in 15 min UV detection at 220nm. B) ESI MS, inserted the molecular structure.

A

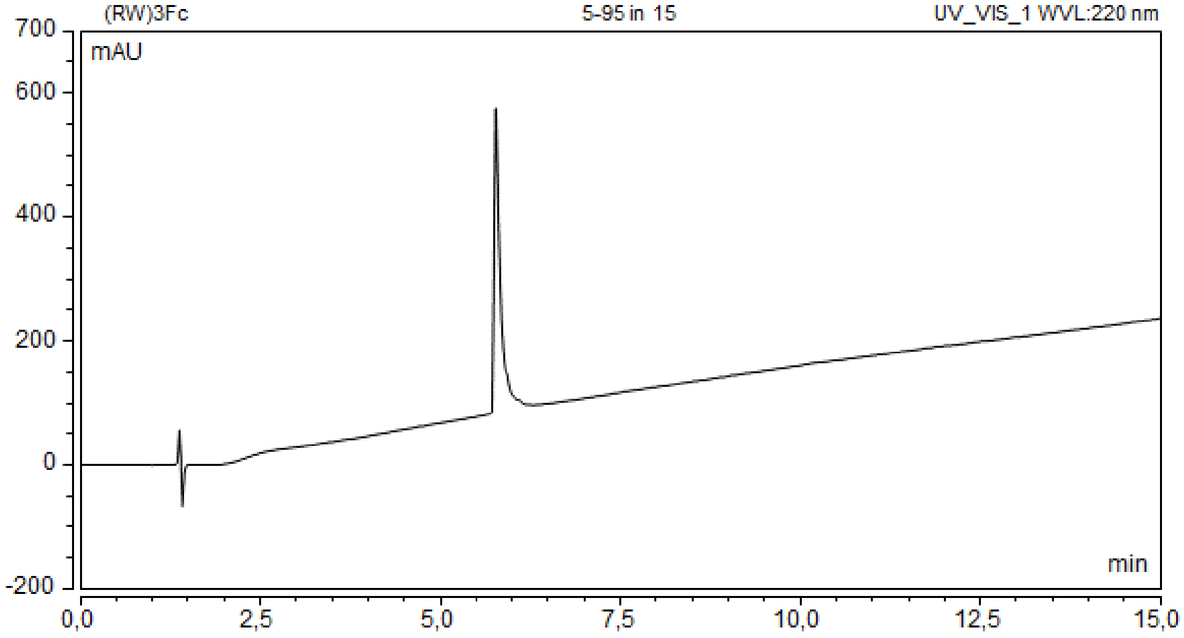

B

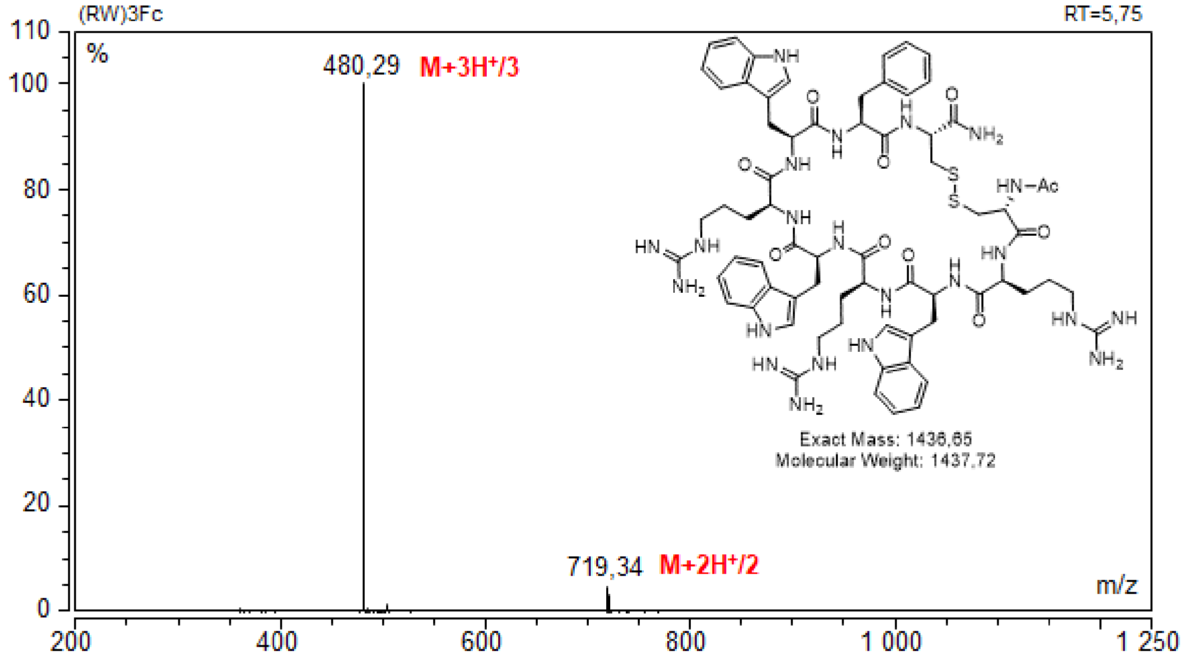

Figure S6: A) HPLC trace of (WR)$_3$Fc. Gradient 5 to 95% B into A in 15 min UV detection at 220nm. B) ESI MS, inserted the molecular structure.

A

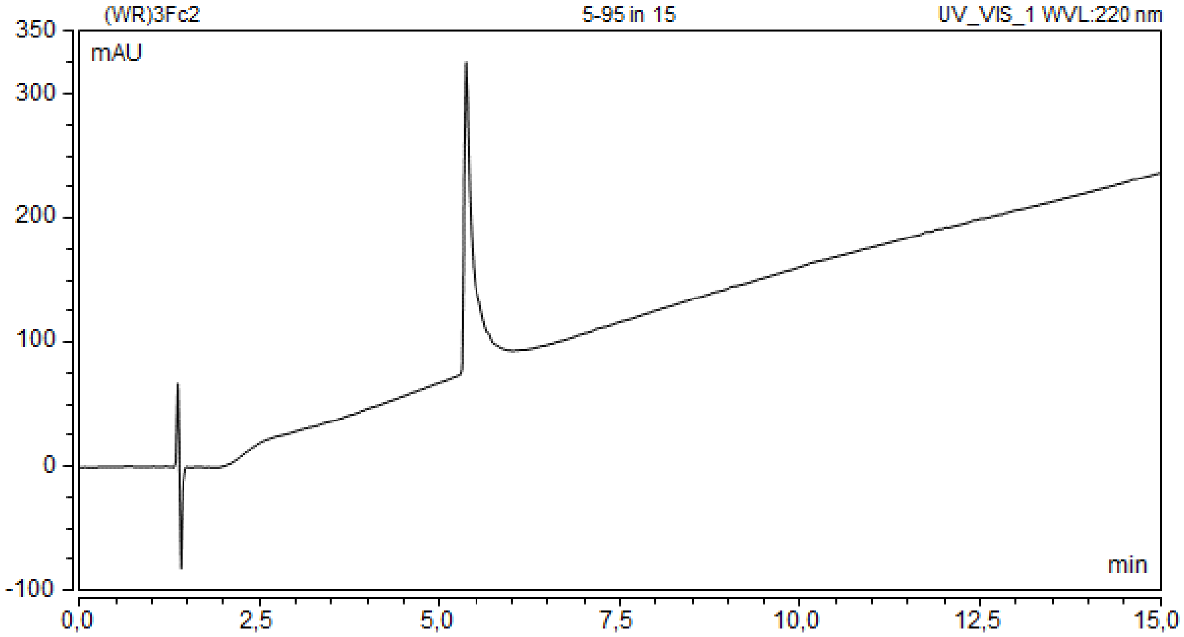

B

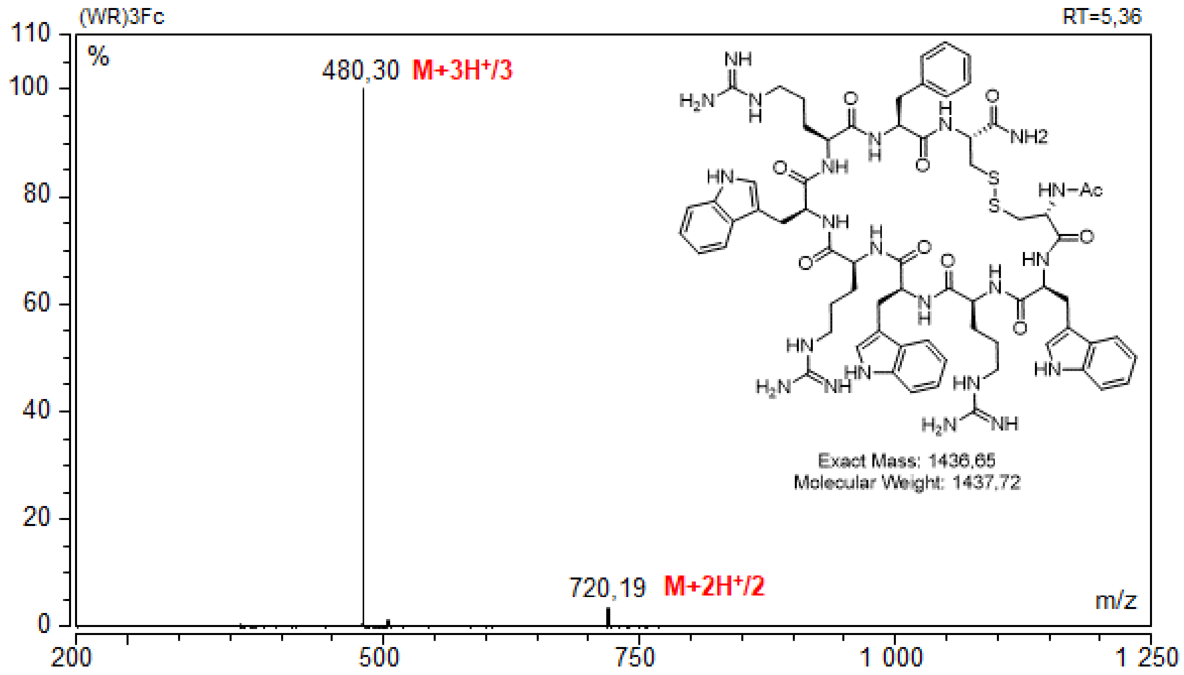

Figure S7: Acridine orange uptake in the presence of efflux pump inhibitors and studied peptides.

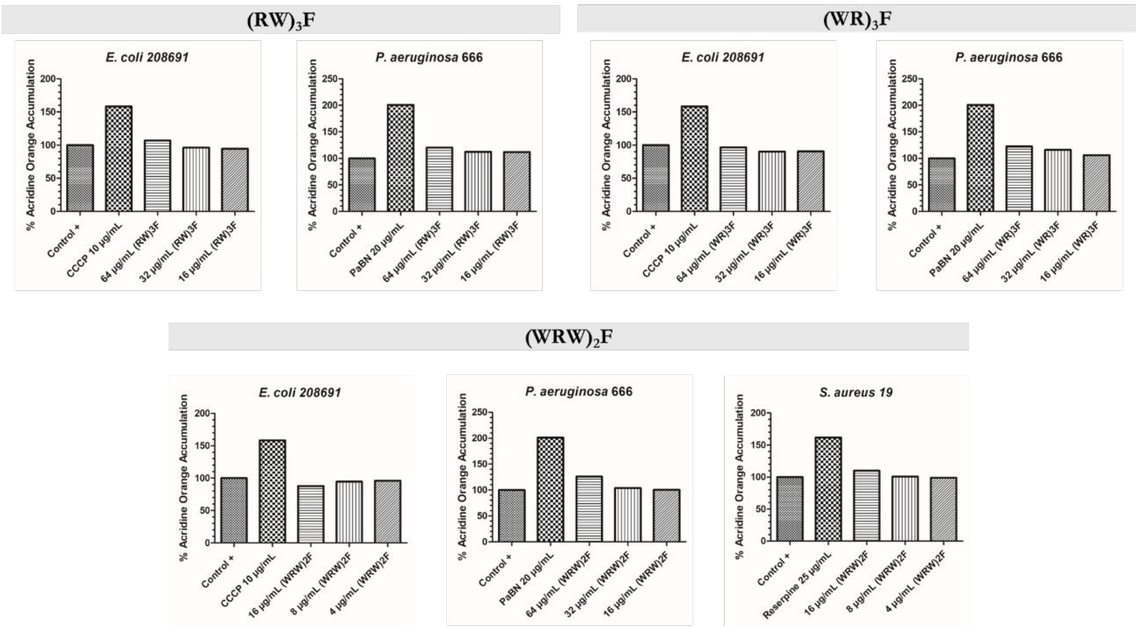

Supplement: Supplementary file 1 [file ao5c12724_si_001.pdf]
